# Antibiotic Exposure in Early Life Increases Risk of Childhood Obesity: A Systematic Review and Meta-Analysis

**DOI:** 10.3389/fendo.2017.00170

**Published:** 2017-07-20

**Authors:** Xiaoqing Shao, Xiaolian Ding, Bin Wang, Ling Li, Xiaofei An, Qiuming Yao, Ronghua Song, Jin-an Zhang

**Affiliations:** ^1^Department of Endocrinology, Jinshan Hospital of Fudan University, Shanghai, China; ^2^Department of Nephrology and Endocrinology, Weinan Central Hospital, Weinan, China

**Keywords:** antibiotics, childhood obesity, meta-analysis, risk factor, overweight

## Abstract

A number of studies have previously assessed the impact of antibiotic exposure in early life on the risk of childhood obesity, but no systematic assessment is currently available. A systematic review and meta-analysis was performed to comprehensively and quantitatively elucidate the risk of childhood obesity caused by antibiotic exposure in early life. Literature search was performed in PubMed, Embase, and Web of Science. Random-effect meta-analysis was used to pool the statistical estimates. Fifteen cohort studies involving 445,880 participants were finally included, and all those studies were performed in developed countries. Antibiotic exposure in early life significantly increased risk of childhood overweight [relative risk (RR) = 1.23, 95% confidence interval (CI) 1.13–1.35, *P* < 0.001] and childhood obesity (RR = 1.21, 95% CI 1.13–1.30, *P* < 0.001). Antibiotic exposure in early life also significantly increased the *z*-score of childhood body mass index (mean difference: 0.07, 95% CI 0.05–0.09, *P* < 0.00001). Importantly, there was an obvious dose–response relationship between antibiotic exposure in early life and childhood adiposity, with a 7% increment in the risk of overweight (RR = 1.07, 95% CI 1.01–1.15, *P* = 0.03) and a 6% increment in the risk of obesity (RR = 1.06, 95% CI 1.02–1.09, *P* < 0.001) for each additional course of antibiotic exposure. In conclusion, antibiotic exposure in early life significantly increases risk of childhood obesity. Moreover, current analyses are mainly taken from developed countries, and therefore the impact of antibiotic exposure on risk of childhood obesity in vulnerable populations or developing countries still needs to be evaluated in future studies.

## Introduction

Obesity has increased significantly among children and adolescents over the past several decades, and childhood adiposity has become a major challenge to public health worldwide (1, 2). In the United States, the prevalence of obesity in children and adolescents is about 17.0% and it has also increased substantially (3). Excess adiposity during childhood is likely to persist into adulthood and further increases the predisposition to cardiovascular diseases, cancer, and early mortality (4, 5). Childhood obesity also increases risk of other diseases during childhood, such as hypertension and depression (6–8). In addition, some obese children have exhibited early signs of cardiovascular dysfunction (9). The pathogenesis of obesity is complex and may involve many factors, and studies that explore the causes of the increasing epidemic of childhood obesity and possible solutions to this issue are necessary (10).

Emerging evidence has suggested that gut microbiota is linked to obesity and other metabolic disorders (11, 12). There are obvious changes in gut microbiota of patients with obesity, and these changes may precede the clinical manifestation of obesity (13). Antibiotics can result in obvious alterations in the gut microbiota and even gut dysbiosis, which may further result in obesity or other metabolic disorders (12, 14, 15). Considering that antibiotics are frequently prescribed in pediatric patients, a precise assessment of the relationship between antibiotics and childhood obesity is very important (16). There are a number of epidemiological studies assessing the impact of antibiotic exposure in early life on childhood adiposity risk (17–24). Several studies reported that antibiotic exposure could increase the risk of childhood overweight or obesity (17, 19, 23, 24), while the other studies reported that antibiotic exposure had no obvious role on childhood adiposity risk (18, 21). Besides, no systematic review and meta-analysis is currently available that provides a definite evaluation of the relationship between antibiotic exposure and childhood adiposity. An improved understanding of the issue has important implications for public health, because there is an increasing epidemic of childhood obesity and prudent use of antibiotics in early life may be one way to reduce this epidemic. Therefore, a systematic review and meta-analysis of available studies was performed to assess the relationship between antibiotic exposure in early life and childhood adiposity.

## Materials and Methods

### Literature Search

A literature search in PubMed, Emabse, and Web of Science was carried out to find articles that examined the impact of antibiotic exposure on childhood adiposity risk. The following terms were used: (antibiotics OR antibiotic OR tetracycline OR doxycycline OR cephalosporin OR penicillin OR metronidazole OR fluoroquinolone OR sulfonamide OR macrolide) AND (adiposity OR obesity OR obese OR overweight OR body mass index) AND (child OR children OR childhood OR boy* OR girl* OR infancy OR infants). All databases were searched from their inception through September 6, 2016, and an updated literature search was performed on February 26, 2017. Language restriction was not applied. Bibliographies of eligible articles and relevant reviews were also screened.

### Selection Criteria

Selection criteria for the meta-analysis were as following: (1) prospective or retrospective cohort studies; (2) the outcomes of interest were the association between antibiotic exposure and childhood adiposity, or the impact of antibiotic exposure on childhood body mass index (BMI) or weight; (3) the exposure time was early life including infancy and prenatal period; (4) reporting risk estimates on the associations, such as relative risks (RRs) with 95% confidence intervals (95% CIs), or the difference in the *z*-scores of childhood BMI or weight between the exposed group and the non-exposed group. Infancy is usually defined as the period from birth to 2 years of age. Early life includes infancy and prenatal period, and is thus from fetal life through 2 years of age in the present study. Studies containing overlapping data, case reports, or case series were all excluded.

### Data Extraction and Quality Evaluation

Two colleagues extracted data independently, and discrepancy was resolved by discussion. The extracted data mainly included first author, country, study design, antibiotic exposure, participants, follow-up time, adjusted variables, and outcomes of interest. If one study reported data of multiple follow-ups, we used the data for the longest follow-up time. The authors of included studies were contacted if crucial information was not provided in the articles.

The primary outcome of interest was the association of antibiotic exposure in early life with childhood overweight or obesity. The secondary outcome of interest was the difference of the *z*-scores of childhood BMI or weight between the exposed group and the non-exposed group. The evaluation of study quality was performed by the Newcastle–Ottawa scale, and studies scoring seven or more points were identified as high-quality studies (25).

### Data Analysis

The risk estimates on the associations between antibiotic exposure and childhood adiposity and the differences of childhood body weight between the exposed group and the non-exposed group were pooled using meta-analysis. Heterogeneity was measured using Cochran-*Q* test and *I*^2^ method (26, 27). Random-effect model was utilized to pool data in order to reduce the impact of heterogeneity across studies (28). Subgroup analyses were carried out by gender, exposure time, and study design. The study by Li et al. (29) reported data from two different comparisons, and we firstly used the risk estimate from the comparison between infants who used antibiotics and those who did not, and then performed a sensitivity analysis by using the risk estimate from the comparison between infants who used antibiotics and those who had infections but did not receive antibiotics. Publication bias was evaluated by Egger’s test and funnel plot (30). For dose–response meta-analysis, the correlated log RR estimates across different courses of antibiotic exposure were calculated using generalized least-square regression method, and RRs were further pooled using random-effect meta-analysis (31, 32). STATA (version 12.0) and Review Manager (version 5.2) were used for statistical analyses. *P* < 0.05 indicated a statistically significant difference.

## Results

### Study Selection and Characteristics

**Figure 1:**
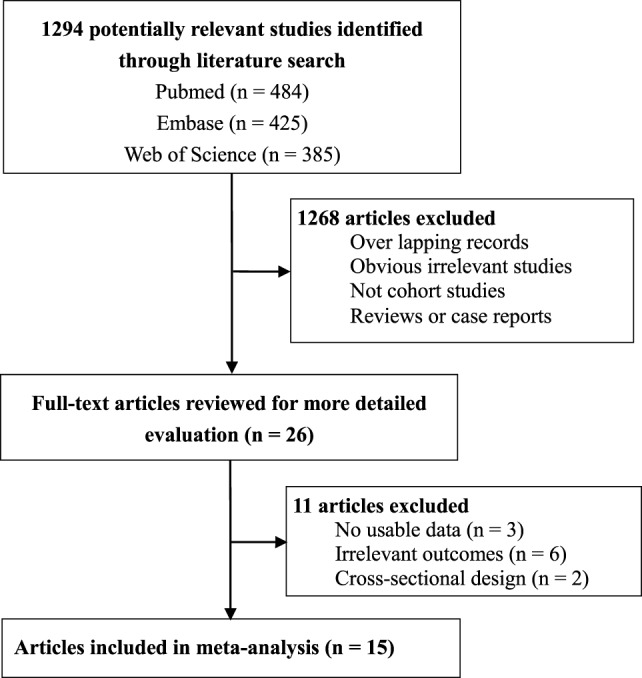
Flow chart of study selection in the meta-analysis.

From the literature search, we found a total of 1,294 abstracts, but only 26 studies were considered as potentially eligible and assessed by reading full-texts (17–24, 29, 33–49) (Figure 1). After detailed evaluation, 11 studies were further removed including 6 studies for irrelevant outcomes (34–36, 40, 42, 47), 3 studies for no usable data (38, 43, 44), and 2 studies for cross-sectional design (37, 46). The study by Korpela et al. only reported the *r*-value from linear correlation analysis, but did not report the difference in the *z*-score of childhood BMI or weight (43). Ultimately, 15 cohort studies involving 445,880 participants were included (17–24, 29, 33, 39, 41, 45, 48, 49) (Figure 1). Table 1 showed the characteristics of those 15 cohort studies (Table 1). There were 12 prospective cohort studies, and 3 retrospective cohort studies (Table 1). Those studies were published from 2011 to 2017. There was an obvious difference in the number of participants, which ranged from 97 to 260,556. All studies were performed in developed countries, and no study was from developing countries or was performed in predefined vulnerable populations (Table 1). Besides, about 40% of the studies were in the United States and represented over 75% of all subjects (Table 1).

**Table 1:** Characteristics of those 15 cohort studies on the association between antibiotic exposure in early life and risk of childhood adiposity.

Study	Location (baseline time)	Study design	Participants	Exposure time	Time of follow-up	Outcomes	Adjustment	Quality^a^
Ajslev et al. (17)	Denmark (1997–2002)	Prospective cohort	28,354 children dyads from the Danish National Birth Cohort	Infancy (<6 months)	7 years	Overweight	Maternal age, socioeconomic status, prepregnancy BMI, gestational weight gain, smoking, paternal BMI, parity, birth weight, sex, breastfeeding, and age at 7-year follow-up	9

Halldorsson et al. (33)	Denmark (1988–1989)	Prospective cohort	665 pregnant women with offspring	Prenatal	20 years	Overweight	Maternal age, maternal education, maternal prepregnancy BMI, smoking during pregnancy, parity, infant birth weight, and offspring age at follow-up	8

Murphy et al. (18)	New Zealand (1995–1996)	Prospective cohort	871 European children	Infancy (<12 months)	11 years	BMI *z*-score	Maternal smoking and breastfeeding	7

Trasande et al. (23)	UK (1991–1992)	Prospective cohort	11,532 children	Infancy (<6 months)	7 years	Overweight, obesity, BMI *z*-score	Birth weight, maternal parity, race, social class, education, parental BMI, first trimester smoking, breastfeeding, timing of introduction of complementary foods, time spent per day watching television, in car on weekdays, in car on weekends, dietary pattern classifications at 38 months, and duration of nighttime sleep at 7 years	9

Bailey et al. (19)	USA (2001–2009)	Prospective cohort	64,580 children	Infancy (<24 months)	5 years	Obesity	Steroid use, gender, age, urban practice, public insurance, primary care visits, Hispanic ethnicity, diagnosed asthma or wheezing, calendar year of first visit, antireflux medication use, and common infectious diagnoses.	9

Azad et al. (24)	Canada (1995)	Prospective cohort	616 children	Infancy (<12 months)	9 years	Overweight	Birth weight, breastfeeding, smoke exposure at birth, family income, sibship, diet, physical activity at age 9, current asthma, maternal asthma, and maternal overweight	8

Saari et al. (41)	Finland (2003–2007)	Prospective cohort	6,114 healthy boys and 5,948 healthy girls	Infancy (<24 months)	More than 2 years	Overweight, BMI *z*-score	Maternal smoking after the first trimester, parental relationship, mode of delivery, birth weight, and birth length	9

Mueller et al. (20)	USA (1998–2006)	Prospective cohort	436 children	Prenatal	7 years	Obesity, BMI *z*-score	Maternal age, ethnicity, pregravid BMI, maternal receipt of public assistance, birth weight, sex, breast feeding in the first year, and gestational antibiotics or delivery model	7

Mor et al. (39)	Denmark (1994–1998)	Prospective cohort	9,886 school children	Prenatal	14.5 years	Overweight, obesity	Maternal age at delivery, marital status, smoking in pregnancy multiple gestation, and birth weight	8

Scott et al. (45)	UK (1995–2003)	Retrospective cohort	1,714 children in The Health Improvement Network	Infancy (<24 months)	4 years	Obesity, BMI *z*-score	Year of birth, maternal and sibling obesity, maternal diabetes, mode of delivery, country of origin, urban environment, and Townsend score	8

Mbakwa et al. (22)	Netherlands (2000–2002)	Prospective cohort	979 children	Infancy (<24 months)	9 years	Overweight, weight *z*-score, BMI *z*-score	Recruitment group, household size, maternal level of education, maternal prepregnancy weight, maternal pregnancy weight gain, smoking during pregnancy, gestational diabetes, gestational hypertension, place, and mode of delivery, sex, birth weight, gestational age, duration of breastfeeding, dietary intake, child’s physical activity, and child’s ages during anthropometric measurements	8

Gerber et al. (21)	USA (2001–2011)	Retrospective cohort	44,737 children	Infancy (<6 months)	5 years	Weight *z*-score	Sex, birth weight, race, Medicaid insurance status, number of siblings, birth year, baseline length, and primary care site	9

Li et al. (29)	USA (1997–2013)	Retrospective cohort	260,556 children	Infancy (<12 months)	9 years	Obesity	Maternal age, race or ethnic origin, prepregnancy BMI, preterm delivery, low birth weight, maternal antibiotic use, and infection during pregnancy	9

Poulsen et al. (49)	USA (2006–2012)	Retrospective cohort	8,793 singleton children	Prenatal; infancy (<12 months)	3 years	BMI *z*-score	Centered child exact age, mother race/ethnicity, cesarean section, birth weight, mother Medical Assistance, smoked during pregnancy, parity, and pregravid BMI	8

Ville et al. (48)	USA (2012–2013)	Prospective cohort	97 Latino children	Infancy (<6 months)	2 years	Obesity	Maternal BMI, birth weight, breastfeeding, infant weight gain, and infant sex	7

*^a^Quality was assessed using NOS method*.

Among those 15 studies, 7 studies assessed the relationship between antibiotic exposure and childhood overweight (17, 22–24, 33, 39, 41), 7 studies assessed the relationship between antibiotic exposure and childhood obesity (19, 20, 23, 29, 39, 45, 48), 7 studies analyzed the influence of antibiotic exposure on childhood BMI (18, 20, 22, 23, 41, 45, 49), and 2 studies assessed the influence of antibiotic exposure on childhood body weight (21, 22) (Table 1). Twelve studies assessed the relationship between antibiotic exposure during infancy and childhood adiposity, and four studies examined the impact of prenatal antibiotic exposure on childhood adiposity (Table 1). Six studies reported data for the dose–response meta-analysis (19, 22, 24, 39, 41, 45). All studies reported adjusted risk estimates, but the confounding factors were different (Table 1). The quality of included studies was shown in Table 1.

### Meta-Analysis

**Figure 2:**
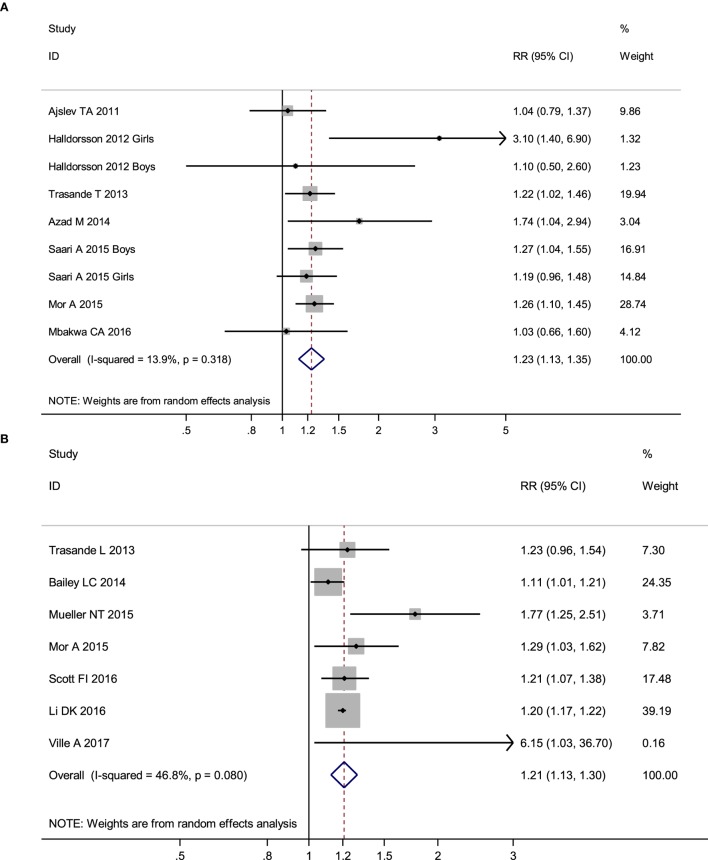

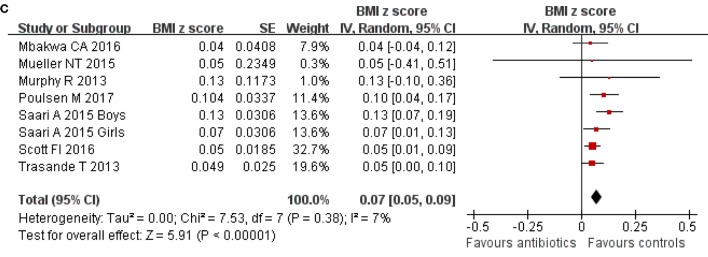
Meta-analysis suggested that antibiotic exposure in early life increased risk of childhood adiposity. **(A)** Meta-analysis of studies on childhood overweight risk associated with antibiotic exposure in early life. **(B)** Meta-analysis of studies on childhood obesity risk associated with antibiotic exposure in early life. **(C)** Meta-analysis of studies on changes of body mass index *z*-scores associated with antibiotic exposure in early life.

No obvious heterogeneity existed in those studies relating antibiotic exposure and childhood overweight (*I*^2^ = 13.9%). Meta-analysis showed that antibiotic exposure in early life (from fetal life through age 2) significantly increased the risk of childhood overweight (RR = 1.23, 95% CI 1.13–1.35, *P* < 0.001) (Figure 2A). Subgroup analysis by exposure time suggested that both antibiotic exposure during infancy (from birth through age 2) and prenatal exposure to antibiotics could increase risk of childhood overweight (Table 2). Other subgroup analyses by gender and time of antibiotic exposure during infancy also found increased risk of childhood overweight among those children with antibiotic exposure in early life (Table 2).

**Table 2:** Meta-analysis of the association between antibiotic exposure in early life and risk of childhood adiposity.

Outcomes	Study (participants)	Pooled estimates (95% CI)	*P*-value	*I*^2^ (%)
**Overweight**				
Total studies	7 (64,096)	1.23 (1.13–1.35)	<0.001	13.9
Prospective cohort studies	7 (64,096)	1.23 (1.13–1.35)	<0.001	13.9
Exposure during infancy	5 (53,545)	1.21 (1.09–1.33)	<0.001	0
Prenatal exposure	2 (10,551)	1.27 (1.11–1.45)	0.001	0
Exposure during infancy (<6 months)	5 (53,545)	1.22 (1.09–1.36)	<0.001	0
Exposure during infancy (6–12 months)	3 (13,659)	1.25 (1.10–1.42)	0.001	0
Exposure during infancy (12–24 months)	2 (13,043)	1.20 (1.01–1.43)	0.042	0
Boys	4 (23,231)	1.48 (1.16–1.88)	0.001	51.4
Girls	4 (23,231)	1.17 (1.02–1.35)	0.029	0
**Obesity**				
Total studies	7 (348,801)	1.21 (1.13–1.30)	<0.001	46.8
Prospective cohort studies	5 (86,521)	1.30 (1.08–1.56)	0.007	63.4
Retrospective cohort studies	2 (262,270)	1.20 (1.18–1.23)	<0.001	0
Exposure during infancy	5 (338,479)	1.18 (1.12–1.25)	<0.001	33.7
Prenatal exposure	2 (10,322)	1.47 (1.08–1.99)	0.014	55.1
Exposure during infancy (<6 months)	5 (338,479)	1.15 (1.04–1.26)	0.005	67.0
Exposure during infancy (6–12 months)	3 (326,850)	1.07 (0.95–1.22)	0.269	83.9
Exposure during infancy (12–24 months)	2 (66,294)	1.06 (0.96–1.16)	0.259	0%
Boys	1 (9,886)	1.29 (0.96–1.73)	0.09	NA
Girls	1 (9,886)	1.27 (0.89–1.82)	0.19	NA
**BMI *z*-score**				
Total studies	7 (36,389)	0.07 (0.05–0.09)	<0.00001	7
Prospective cohort studies	5 (25,882)	0.07 (0.04–0.10)	<0.00001	6
Retrospective cohort studies	2 (10,507)	0.07 (0.02–0.12)	0.008	53%
Exposure during infancy	6 (35,953)	0.07 (0.05–0.10)	<0.00001	20
Boys	1 (12,064)	0.13 (0.07–0.19)	<0.0001	NA
Girls	1 (12,064)	0.07 (0.01–0.13)	0.02	NA
**Weight *z*-score**				
Total studies	2 (45,716)	0.06 (0.01–0.11)	0.03	0

*NA, not available*.

**Figure 3:**
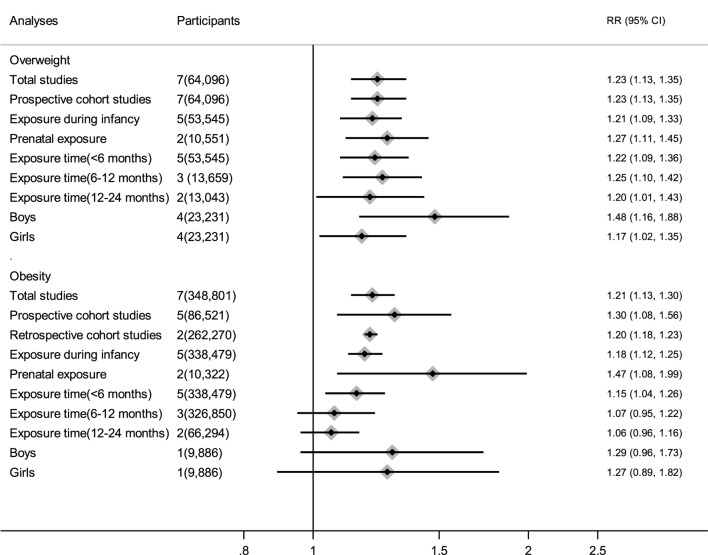
Main findings in the subgroup analyses of the meta-analysis.

There was moderate heterogeneity among studies relating antibiotic exposure and childhood obesity (*I*^2^ = 46.8%). Meta-analysis showed that antibiotic exposure in early life (from fetal life through age 2) also independently increased childhood obesity risk (RR = 1.21, 95% CI 1.13–1.30, *P* < 0.001) (Figure 2B). When using the risk estimate of the comparison between infants who used antibiotics and those who had infections but did not receive antibiotics in the study by Li et al. (29), antibiotic exposure in early life still increased childhood obesity risk (RR = 1.18, 95% CI 1.05–1.32, *P* = 0.005). Subgroup analysis by exposure time suggested that antibiotic exposure during either infancy or prenatal period both increased childhood obesity risk (Table 2; Figure 3).

No heterogeneity existed in those studies relating antibiotic exposure and childhood BMI *z*-score (18, 20, 22, 23, 41, 45, 49) (*I*^2^ = 7%). Meta-analysis showed that antibiotic exposure in early life could dramatically increase the *z*-score of childhood BMI (mean difference: 0.07, 95% CI 0.05–0.09, *P* < 0.00001) (Figure 2C). Antibiotic exposure could markedly increase the *z*-score of childhood BMI in both boys and girls (Table 2; Figure 3).

Meta-analysis of two studies (21, 22) relating antibiotic exposure during infancy and childhood weight showed that antibiotic exposure could significantly increase the *z*-score of childhood weight (mean difference: 0.06, 95% CI 0.01–0.11, *P* = 0.03; *I*^2^ = 0%).

**Figure 4:**
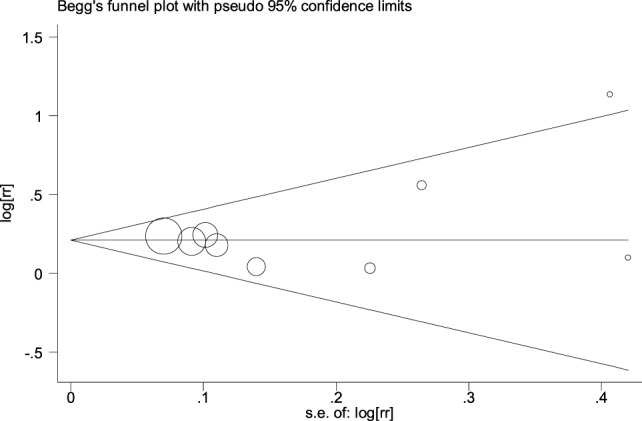
Funnel plot in the meta-analysis on the association between antibiotic exposure in early life and childhood overweight.

The funnel plot did not show an obvious risk of publication bias (Figure 4). *P*_Egger’s test_ was 0.99 and it also proved the lack of publication bias. In addition, funnel plots in other meta-analyses on childhood obesity or childhood BMI also did not show an obvious risk of publication bias.

### Dose–Response Meta-Analysis

**Figure 5:**
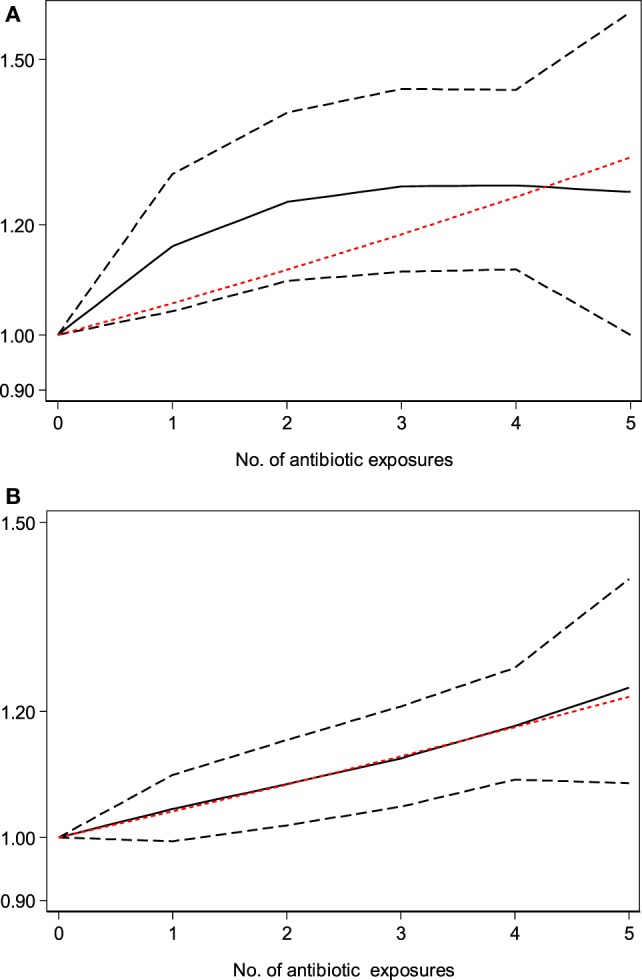
Dose–response meta-analysis of the association between antibiotic exposure in early life and childhood adiposity (the solid black line and the short dash black line represent the estimated relative risk and corresponding 95% confidence intervals of the non-linear relationship; long dash red line represents the linear relationship). **(A)** Dose–response meta-analysis of the association between antibiotic exposure in early life and childhood overweight. **(B)** Dose–response meta-analysis of the association between antibiotic exposure in early life and childhood obesity.

Dose–response meta-analysis of six studies (19, 22, 24, 39, 41, 45) showed that there was an obvious dose–response relationship between antibiotic exposure and childhood adiposity, with a 7% increment in the risk of childhood overweight (per one course RR = 1.07, 95% CI 1.01–1.15, *P* = 0.03) and a 6% increment in the risk of childhood obesity (per one course RR = 1.06, 95% CI 1.02–1.09, *P* < 0.001) for each additional course of antibiotic exposure (Figures 5A,B).

## Discussion

The present study is the first systematic review comprehensively evaluating the impact of antibiotic exposure on childhood adiposity. Fifteen cohort studies involving 445,880 participants were finally included (17–24, 29, 33, 39, 41, 45, 48, 49). The results suggested that antibiotic exposure during early life significantly increased risk of childhood overweight and obesity, and there was an obvious dose-dependent relationship. In addition, antibiotic exposure also significantly increased the *z*-scores of childhood BMI and body weight. Thus, the meta-analysis provides strong evidence for the impact of antibiotic exposure during early life on childhood adiposity risk.

Another important issue is whether antibiotic exposure is an independent risk factor for childhood adiposity. In present meta-analysis, all included studies provided risk estimates adjusted for confounding factors (Table 1). The pooled adjusted RRs for the associations of antibiotic exposure during early life with childhood overweight and obesity were both statistically significant (Table 2). In addition, the *z*-scores of childhood BMI or body weight from included studies were also adjusted for confounding factors, and the pooled *z*-scores were statistically significant (Table 2). Therefore, this meta-analysis proves that antibiotic exposure is an independent risk factor of childhood obesity.

The pathogenesis of obesity is complex and has not been well understood, and it is the same with childhood obesity (50, 51). The role of gut microbiota in the pathogenesis of obesity is increasingly recognized, and accumulating evidence has suggested that gut microbiota has a causal role in obesity (50, 52, 53). Ussar et al. proposed that obesity was a result of interactions between gut microbiota, host genetics, and diet (54). However, the molecular mechanisms underlying the role of gut microbiota in obesity are not yet well known (55). More studies are still needed to further characterize the molecular mechanism of gut microbiota in modulating energy balance and obesity (56). Exploring the role of gut microbiota in childhood obesity may also provide effective prevention or treatment strategies for childhood obesity (57). For example, a recent randomized controlled trial by Nicolucci et al. reported that probiotics can improve the eco-system imbalance of gut microbiotia and reduce the BMI *z*-score in children with overweight or obesity (58).

Data from animal studies have shown that antibiotic-induced changes in gut microbiota can result in fat accumulation by changing host metabolism (59). Antibiotics can also increase insulin resistance by changing gut microbiota (60, 61). Cho et al. reported that antibiotics could alter the murine colonic microbiome and result in the development of obesity (62). Therefore, the important roles of gut microbiota in modulating energy balance and the alerted composition of gut microbiota caused by antibiotics provide some explanations for the association between antibiotic exposure and childhood adiposity. However, more researches are still needed to further explore the complex mechanism underlying this association.

The increasing prevalence of childhood adiposity has become a major global health challenge, and actions to reduce the prevalence of obesity in children and adolescents are urgently needed (4, 63, 64). Investigating risk factors associated with childhood obesity can help us to develop effective prevention interventions (65). The meta-analysis suggests that antibiotic exposure can result in a substantially increased risk of childhood obesity. The finding has important implications in the clinical setting, since antibiotics are the most common drugs administered in infants. Clinicians should weigh the risk of subsequent childhood obesity associated with antibiotic exposure in early life when considering antibiotics in the absence of a clear indication. The meta-analysis also indicates that prudent use of antibiotics may help to minimize the metabolic consequences of antibiotic exposure in early life. In addition, the approach that uses population analyses to understand pharmacokinetic/pharmacodynamic (PK/PD) parameters in children exposed to antibiotics is helpful to define optimal antibiotic dose and course duration, which undoubtedly can improve clinical outcomes and patient safety (66). To assess the impact of antibiotic exposure in early life on childhood obesity more adequately, population PK/PD analyses exploring the impact of antibiotics in different doses and course duration on body composition in children are recommended in future research.

Apart from the prudent use of antibiotics in infants, it is also important for us to develop some plausible methods to prevent obesity in those infants or children who have to receive the treatment of antibiotics. Several studies have studied some possible ways to prevent obesity in children who expose to antibiotics. Kaliannan et al. demonstrated a beneficial effect of omega-3 fatty acid in preventing antibiotic-induced gut dysbiosis and obesity (67). Economopoulos et al. found that co-administration of intestinal alkaline phosphatase with azithromycin prevented the development of metabolic diseases in mice, and the effect was mediated by alterations in gut microbiota (68). However, more studies are still needed to develop some effective methods to prevent the antibiotics-induced obesity in infants or children exposing to antibiotics.

The meta-analysis had several strengths. Firstly, the large number of participants could provide a precise evaluation of the relationship between antibiotics and childhood adiposity. In addition, the obvious dose-dependent relationship strengthened the evidence for the association. Finally, the consistency in the findings of several different types of outcomes further supported the robustness of the findings.

There were several limitations in the meta-analysis. Firstly, all of the current studies were taken from developed countries and there were missing data from developing countries or vulnerable populations. The current study is representative of developed countries, whereas the impact of antibiotics in children in vulnerable populations or developing countries may likely be different from that found in developed countries. Therefore, the impact between antibiotic exposure and childhood adiposity in developing countries or vulnerable populations is still unclear, and needs to be evaluated in future research. Secondly, there were obvious differences in those included studies. The confounding factors used in the adjusted estimates were different, which could result in heterogeneity and different outcomes (69). Thirdly, the use of antibiotics during follow-up may be an important factor modifying the impact of antibiotics on childhood adiposity risk. However, most included studies did not add antibiotics use during follow-up as a confounding factor, which might cause risk of bias. Fourthly, there were different types of antibiotics among included studies, and they might have different impact on childhood adiposity. Further studies are required to explore the impact of different types of antibiotics on childhood obesity. Finally, the relationship between antibiotic exposure and adiposity in adults has not been studied, and future research is recommended.

In summary, this meta-analysis provides strong evidence for the association between antibiotic exposure in early life and childhood adiposity. There is also an obvious dose–response relationship between antibiotic exposure and childhood adiposity. Prudent use of antibiotics is recommended for infants and children to reduce the risk of childhood adiposity. Future studies are also required to develop some effective methods to prevent obesity in infants or children exposing to antibiotics.

## Author Contributions

XS and JZ designed the study. XS, BW, XD, RS, and JZ contributed to the literature search, interpretation, writing, and proofreading of the manuscript. XS, BW, LL, and QY extracted data and performed data analyses. XS, BW, and XA revised the study. XS and BW generated the figures.

## Conflict of Interest Statement

The authors declare that the research was conducted in the absence of any commercial or financial relationships that could be construed as a potential conflict of interest.
